# Supplementary material for: The Impact of the first COVID-19 shelter-in-place announcement on social distancing, difficulty in daily activities, and levels of concern in the San Francisco Bay Area: A cross-sectional social media survey
Source: PLoS One. 2021 Jan 14;16(1):e0244819. doi: 10.1371/journal.pone.0244819 (PMC7808609; doi:10.1371/journal.pone.0244819)
Supplement: S1 Fig — We depict the frequency of survey responses in the Bay Area and elsewhere in the U.S. by date (top panel); cumulative survey responses by date as percentages (middle panel); and the timing of statewide shelter-in-place orders (bottom panel). (DOCX) [file pone.0244819.s001.docx]

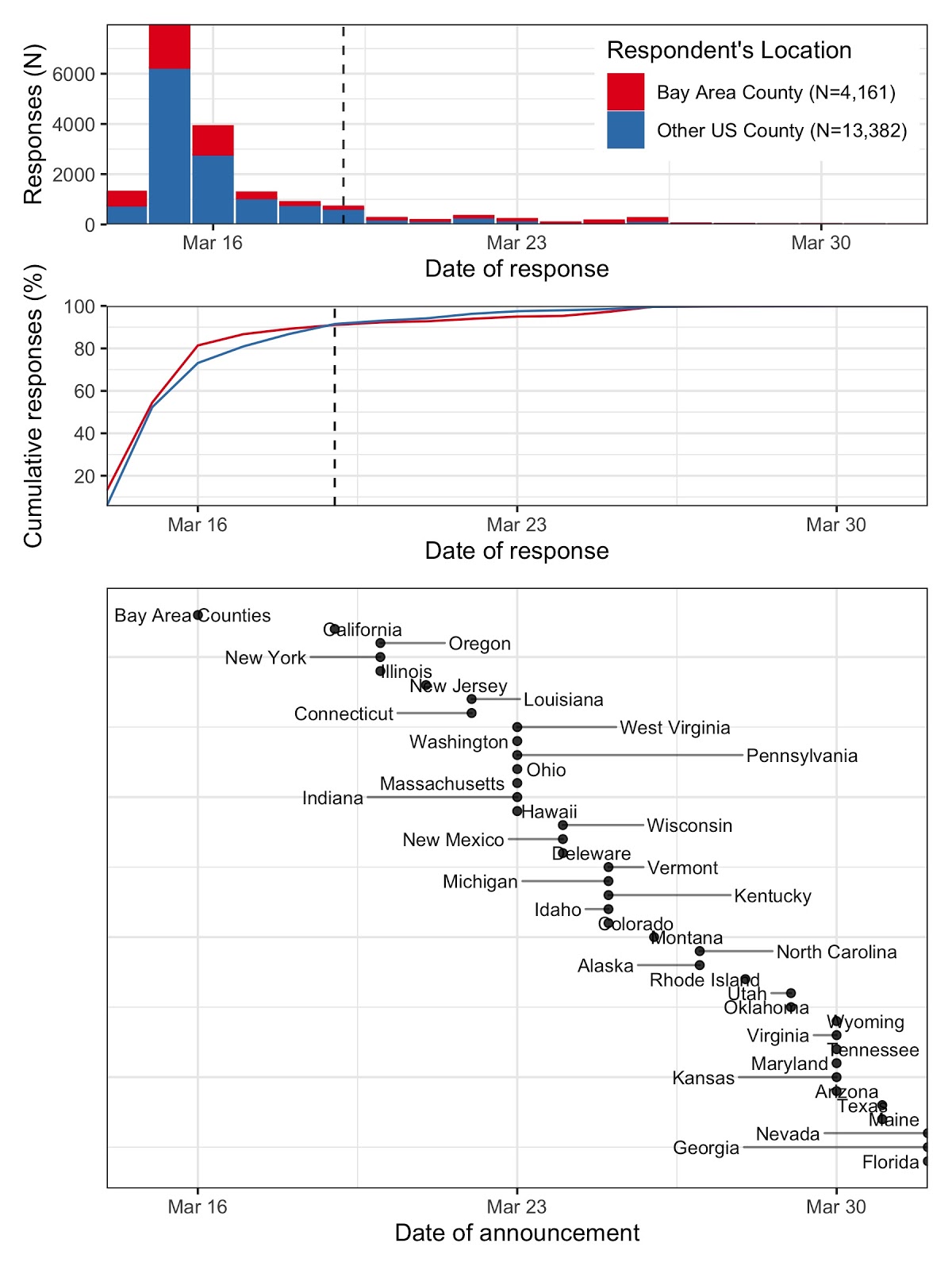


**Figure 1. Timing of survey responses and statewide shelter-in-place orders.**

We depict the frequency of survey responses in the Bay Area and elsewhere in the U.S. by date (top panel); cumulative survey responses by date as percentages (middle panel); and the timing of statewide shelter-in-place orders (bottom panel)
