# Supplementary material for: The Impact of the first COVID-19 shelter-in-place announcement on social distancing, difficulty in daily activities, and levels of concern in the San Francisco Bay Area: A cross-sectional social media survey
Source: PLoS One. 2021 Jan 14;16(1):e0244819. doi: 10.1371/journal.pone.0244819 (PMC7808609; doi:10.1371/journal.pone.0244819)
Supplement: S1 Table — (DOCX) [file pone.0244819.s002.docx]

**Supplemental Table 1.** Demographics for the Bay Area and other U.S. states before and after the March 16, 2020 shelter-in-place announcement – N (%) **^1^**

|  | **Bay Area ^2^** | | **Elsewhere** | |
| --- | --- | --- | --- | --- |
|  | **Before** (N = 2,951) | **After** (N = 1,210) | **Before** (N = 8,410) | **After** (N = 4,972) |
| **Gender**  Female  Male  Other | 2,197 (74.2)  747 (25.2)  16 (0.5) | 929 (76.8)  268 (22.1)  11 (0.9) | 4,419 (52.5)  2,774 (33.0)  94 (1.1) | 3,931 (79.1)  983 (19.8)  48 (1.0) |
| **Race/Ethnicity ^3^**  Non-Hispanic White  Asian and Pacific Islander  Hispanic/Latino  Black  Other | 2,185 (73.8)  452 (15.3)  137 (4.6)  15 (0.5)  116 (3.9) | 878 (72.6)  177 (14.6)  67 (5.5)  16 (1.3)  52 (4.3) | 7,137 (84.9)  154 (1.8)  358 (4.3)  117 (1.4)  262 (3.1) | 4,366 (87.8)  154 (3.1)  186 (3.7)  70 (1.4)  144 (2.9) |
| **Age**  < 25 years  26 – 35 years  36 – 45 years  46 – 55 years  56 – 65 years  > 65 years | 84 (2.8)  451 (15.2)  752 (25.4)  695 (23.5)  517 (17.5)  423 (14.3) | 52 (4.3)  290 (24.0)  277 (22.9)  230 (19.0)  198 (16.3)  155 (12.8) | 600 (7.1)  1,802 (21.4)  2,319 (27.6)  1,803 (21.4)  1,224 (14.6)  630 (7.5) | 276 (5.6)  1,191 (24.0)  1,374 (27.6)  933 (18.8)  649 (13.1)  526 (10.6) |
| **Education**  Less than High School  High School or GED  Some College  Bachelor’s Degree | 5 (0.2)  34 (1.1)  277 (9.4)  2,633 (88.9) | 3 (0.2)  19 (1.6)  134 (11.1)  1,049 (86.7) | 20 (0.2)  214 (2.5)  1,259 (15.0)  6,914 (82.2) | 19 (0.4)  108 (2.2)  771 (15.5)  4,070 (81.9) |
| **Health Insurance**  Yes  No  I don’t Know | 2,905 (98.1)  31 (1.0)  8 (0.3) | 1,180 (97.5)  27 (2.2)  2 (0.2) | 8,047 (95.7)  327 (3.9)  18 (0.2) | 4,785 (96.2)  163 (3.3)  14 (0.3) |
| **Children in Household (<18 years)**  None  One  Two  Three or more | 1,547 (52.2)  471 (15.9)  734 (24.8)  171 (5.8) | 749 (61.9)  178 (14.7)  201 (16.6)  74 (6.1) | 5,005 (59.5)  1,315 (15.6)  1,420 (16.9)  592 (7.0) | 2,981 (60.0)  759 (15.3)  825 (16.6)  361 (7.3) |
| **Senior in Household (>65 years)**  None  One  Two  Three or more | 2,293 (77.4)  432 (14.6)  178 (6.0)  19 (0.6) | 929 (76.8)  188 (15.5)  72 (6.0)  5 (0.4) | 6,920 (82.3)  955 (11.4)  412 (4.9)  31 (0.4) | 4,118 (82.8)  573 (9.5)  202 (4.1)  21 (0.4) |

**1.** Gender was missing for 44 respondents; race/ethnicity was missing for 203 respondents; age is missing for 92 respondents; educational attainment was missing for 14 respondents; health insurance status was missing for 36 respondents; number of children (< 18 years) in the household was missing for 160 respondents and number of seniors (> 65 years) in household was missing for 195 respondents.

**2.** Respondents in the Bay Area included those who resided in San Francisco, Santa Clara, San Mateo, Marin, Contra Costa, Alameda, or Santa Cruz county at the time they completed the survey. Respondents elsewhere were those who resided in other California counties or other U.S. states. International respondents were excluded.

**3.** Asian and Pacific Islander includes respondents who identified as Asian Indian, Chinese, Japanese, Korean, Vietnamese, Filipino, Native Hawaiian, Chamorro, other Pacific Islander, or other Asian.
