# Supplementary material for: The Impact of the first COVID-19 shelter-in-place announcement on social distancing, difficulty in daily activities, and levels of concern in the San Francisco Bay Area: A cross-sectional social media survey
Source: PLoS One. 2021 Jan 14;16(1):e0244819. doi: 10.1371/journal.pone.0244819 (PMC7808609; doi:10.1371/journal.pone.0244819)
Supplement: S2 Table — (DOCX) [file pone.0244819.s003.docx]

**Supplemental Table 2.** DID estimates for experienced difficulties in California versus elsewhere following the March 16, 2020 announcement of the Bay Area shelter in place order **^1^**

|  | **ß (95% CI)** **^2^** |
| --- | --- |
| **Food** | 5.15 (1.82, 8.48) |
| **Transportation** | 2.21 (- 1.46, 5.89) |
| **Healthcare** | 1.38 (0.02, 2.74) |
| **Hand Sanitizer** | 0.36 (- 1.31, 2.03) |
| **Medication** | - 0.18 (- 2.20, 1.83) |
| **Job Loss** | - 0.38 (- 1.37, 0.60) |
| **Childcare ^3^** | - 1.72 (- 4.24, 0.81) |
| **Wages** | - 1.99 (- 7.37, 3.39) |

1. Participants were asked to select all difficulties they were experiencing during the COVID-19 crisis.
2. We used linear probability models to estimate the change in California versus elsewhere for each of the above experienced difficulties and transformed model coefficients into percentages by multiplying estimated proportions by 100%.
3. Analysis restricted to survey respondents with at least one child under the age of 18 in their household (N = 7,062)
